# Men’s Responses to Online Smoking Cessation Resources for New Fathers: The Influence of Masculinities

**DOI:** 10.2196/resprot.4079

**Published:** 2015-05-13

**Authors:** Joan L Bottorff, John L Oliffe, Gayl Sarbit, Mary Theresa Kelly, Alexandra Cloherty

**Affiliations:** ^1^Institute for Healthy Living and Chronic Disease PreventionUniversity of British ColumbiaKelowna, BCCanada; ^2^Faculty of Health SciencesAustralian Catholic UniversityMelbourneAustralia; ^3^School of NursingFaculty of Applied ScienceUniversity of British ColumbiaVancovuer, BCCanada

**Keywords:** Cancer prevention, smoking cessation, gender, men’s health promotion, fathers, oncology

## Abstract

**Background:**

Smoking cessation is the single most important step to preventing cancer. Drawing on previous research, Web-based resources were developed to complement a program to support expectant and new fathers to quit smoking.

**Objective:**

The objectives of this research were to: (1) describe the responses of expectant and new fathers who smoke or had recently quit smoking to the website resources, and (2) explore how masculinities shape men’s responses to and experiences with online smoking cessation resources.

**Methods:**

Using semi-structured, individual face-to-face interviews, the Dads in Gear Web-based resources were reviewed and evaluated by 20 new fathers who smoked or had recently quit smoking. The data were transcribed and analyzed using NVivo 8 qualitative data analysis software.

**Results:**

We describe the fathers’ reactions to various components of the website, making connections between masculinities and fathering within 5 themes: (1) Fathering counts: gender-specific parenting resources; (2) Measuring up: bolstering masculine identities as fathers; (3) Money matters: triggering masculine virtues related to family finances; (4) Masculine ideals: father role models as cessation aids; and (5) Manly moves: physical activity for the male body.

**Conclusions:**

A focus on fathering was an effective draw for men to the smoking cessation resources. The findings provide direction for considering how best to do virtual cessation programs as well as other types of online cancer prevention programs for men.

## Introduction

Tobacco use remains one of the leading causes of cancer death among men [1-3]. The links between smoking and cancer are irrefutable, and secondhand smoke is also a proven cause of lung cancer in nonsmoking adults [1-2]. Smoking cessation (SC) programs are the most cost-effective interventions to decrease cancer incidence, and there is growing evidence that gender-specific and gender-sensitive approaches can promote SC [4]. There is also a recognized need for men-friendly health promotion interventions that mobilize positive aspects of masculinities and gender relations to enhance men’s well-being [5,6]. However, a systematic review of SC programs targeting men revealed that few men-specific SC interventions exist [7].

Men’s smoking decreases their partners’ success in quitting smoking and maintaining a quit during pregnancy and the postpartum period [8-11], negatively impacts the health of their children [12-14], and triples the chances of their children smoking [15]. Becoming a father is a significant life transition, which challenges men to reconcile their protector and provider roles with continued smoking [5,8,16-17]. To maximize SC when men’s aspirations to be good fathers and role models for their children are at odds with smoking, we designed a targeted 8-week group program, Dads in Gear (DIG) [4]. The DIG program uses men-friendly approaches to integrate SC support, fathering skills, and healthy living (ie, physical activity and healthy eating) to increase the success of quitting. This novel approach drew on our research findings [16,18-20] and participants’ suggestions that peer support was key to SC.

**Figure 1:**
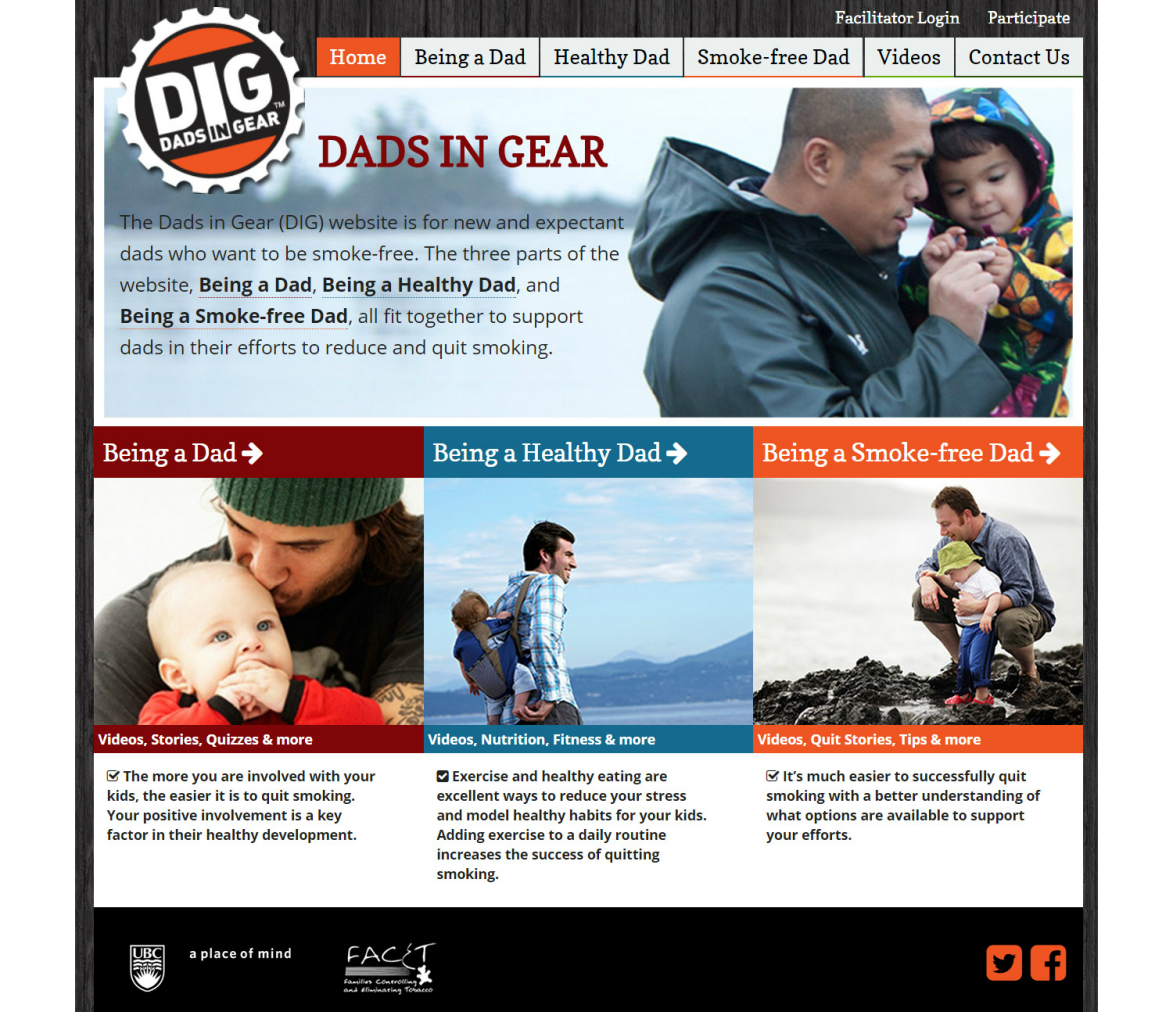
Dads in Gear website [25].

Although the focus of the DIG program facilitates peer support in a face-to-face group format, emergent literature suggests that integrating Web-based technologies can aid feasibility and increase accessibility and dissemination of men’s health promotion programs [21-24]. Accordingly, a suite of online resources were developed to augment and supplement the DIG program in order to: (1) offer easily accessible resources, (2) provide content to support and sustain men’s self-management, and (3) facilitate an online community of fathers who want to quit smoking (see Figure 1).

The three focus areas of the DIG website were smoking cessation (eg, Being a Smoke-free Dad), fathering (eg, Being a Dad), and healthy living (eg, Being a Healthy Dad). The resources affirmed fathering and included a variety of avenues toward SC, avoiding the stigma, guilt, and shame associated with parental smoking. Included among the resources were YouTube-style videos that incorporated fathers’ testimonials about quitting smoking; interactive quizzes related to fathering, fitness, and smoking; infographics that translated information on a variety of topics, including managing cravings, healthy eating, and the benefits of exercise; and a webpage for fathers to share their own stories. The resources were initially reviewed by experts in men’s health, smoking cessation, and Web-based technologies, and refined based on their feedback. The purpose of the current research and this article is to: (1) describe the responses of expectant and new fathers who smoke or had recently quit smoking to the online DIG resources and, (2) explore how masculinities shape men’s responses and experiences to online SC resources.

## Methods

### Recruitment

The DIG website resources were pilot-tested with 20 expectant and new fathers who were interested in quitting smoking or who had recently quit. The study took place in 2 urban centers in British Columbia, Canada. Following ethics approval, fathers were recruited using advertisements on social media (eg, Twitter, Facebook), online media outlets, and printed flyers in community settings. Participants’ demographic characteristics are included in Table 1.

Semi-structured, 3-hour long, individual, face-to-face interviews were conducted by 2 researchers, 1 acting as a facilitator and 1 as note-taker. Following written consent, the fathers completed a short questionnaire to collect data on smoking patterns and demographics, and then engaged in 15 minutes of self-directed browsing of the DIG website. They were asked to “think aloud” as they looked through the website and completed online activities. Field notes were used to capture fathers’ nonverbal behaviors and engagement with the website. The fathers then completed a set of directed tasks and responded to questions about the efficacy, appeal, and usability of the website resources. Finally, open-ended questions gathered men’s perceptions of their overall experiences using the website. The men were provided with an honorarium of $150 CAD. The interviews were digitally recorded, transcribed, and reviewed for accuracy. The field notes were integrated into the transcriptions to contextualize the data.

**Table 1:** The participants’ demographic characteristics and smoking history.

Demographic characteristics		No. of participants (N=20)
**Age range, y (mean=33 y)**	
	20-29	8
	30-39	5
	40-49	4
	50-59	1
	Unknown	2
**Ethnicity**		
	Euro-Canadian	16
	First Nation	2
	Other	2
**Education**		
	Incomplete high school	5
	High school	5
	Postsecondary	9
	Other	1
**Employment**	
	Working	12
	Not working	7
	Student	1
**Marital status**		
	Married	5
	Common-law	11
	Single	4
**Parental status**		
	Have children	19
	Expecting first child	1
	Avg age of youngest child, years	4.54 y
**Smoking history**	
	Mean age started smoking, years	16.5 y
	Mean cigarettes/day when smoking	9.3

### Data Analysis

The analyses involved a close reading of the data by the research team to identify the prevailing meanings, experiences, and views of the fathers. Through an iterative process of discussion and in-depth review of the data, the team developed an analytical framework that delineated major categories and subcategories [26]. The coding schedule derived from this process was used by individual team members to code initial transcripts and, based on further discussion, consensus was reached on minor revisions to refine the framework. All the data were then coded using NVivo 8 qualitative data analysis software. Data segments that were coded to each category were then reviewed, compared, and examined using a gender lens to identify patterns, meanings, and themes. Critical reflection throughout the analyses generated rich and nuanced findings.

## Results

### Overview

Overall the participants responded positively to the DIG website and Web 2.0 resources. The men presented themselves as wanting to be good fathers and showed great interest in fathering and “being a dad,” healthy living, and strategies and tools for reducing and quitting smoking. Connections between masculinities and fathering are reflected in 5 themes describing men’s reactions to the various components of the website: (1) Fathering counts: gender-specific parenting resources; (2) Measuring up: bolstering masculine identities as fathers; (3) Money matters: triggering masculine virtues related to family finances; (4) Masculine ideals: father role models as cessation aids; and (5) Manly moves: physical activity for the male body.

### Fathering Counts: Gender-Specific Parenting Resources

The DIG home page attracted men with the promise of information specific to their interests in being good fathers. Generally confident in their fathering ability, the participants also expressed uncertainty about their knowledge. A 25-year-old father of an infant wondered if he really was the great dad that he aspired to be, saying, “I’m always going, ‘Am I a good dad?’ I wanna be a good dad. How good of a dad am I?”

The participants gravitated toward learning content that provided new activities for involvement with their kids and for keeping their kids healthy and safe. Several men indicated that being able to access fathering information on their own was important so that they did not need to rely on their female partners. A 25-year-old father of 3 stated, “I don’t really like having to go to my kids’ mom to ask her things, ’cause then I kinda feel like I’m not as good of a parent as her.” This father believed he was healthy despite smoking a pack of cigarettes a day, and though he skipped over the health-related content, he was enthusiastic about the variety of resources for fathers. He said, “It wouldn’t really matter what kind of a person you were, or what your interests were . . . there’s something [here] for any dad.”

In contrast to gender norms promulgating the notion that men are unconcerned about nutrition [27], with few exceptions the men genuinely appreciated the cooking and nutrition segments. Participants actually lobbied for more nutrition information and quick healthy recipes. A 28-year-old father, who smoked a pack a day and was raising his 4 children alone, talked about his desire for better nutrition and how the DIG website had already provided him with new healthy food information:

I’m by myself, so I always need something new to cook, right? Cause the kids, you can’t just keep feeding them the same thing all the time. And the different nutrition . . . like the different colors. . . . I didn’t really know that.

A 22-year-old father, one of the youngest and lightest smokers in the study, also responded enthusiastically after viewing the cooking and nutrition segments:

I wanna be a healthy father, and I know a lot of kids are picky on what to eat and it’s really hard to get them to have certain nutrients and vitamins in their food. So blending up some soup with, like, peas and ginger, those are both really healthy. . . . I learned quite a bit, like the more colors, the more nutrition you get. That can make a healthier eating family.

A 41-year-old lone parent of 3, who quit smoking a few years ago, endorsed the Healthy Dad section of the website, stating that he would go back to it to try out the recipes. He positioned himself as a “passionate” father, and redefined domestic work as a masculine project whereby competence was capital within the context of fathering:

I’m a stay-at-home dad, or a full-time dad, whatever you wanna call it. Perhaps a father that’s passionate about being a father would come back for this resource again and again.

Only 1 man—a 55-year-old father of 5 who had smoked for 35 years and had no intention of quitting—criticized the DIG website as more suitable for “moms.” He separated himself from images of fatherhood amid feminizing domestic responsibilities that he perceived as counter to traditional masculine ideals—practices he espoused as being features of a “typical guy.” Nevertheless, he responded positively to the Cooking Pea Soup video featuring a proficient male chef (and father), saying, “Now I could see this [making soup] ’cause I like cooking.” Here cooking was aligned with expertise and choice, which draws on traditional male ideals of autonomy and control.

### Measuring Up: Bolstering Masculine Identities As Fathers

The DIG website included interactive quizzes and polls related to smoking cessation, fathering, and healthy living. Most participants enjoyed completing the quizzes and comparing their knowledge with other fathers. Some men stated that the quizzes and polls were the best part of the website. A 33-year-old father of 3 suggested:

I have an interest in what other dads [are saying] and what the statistics are, basically how I relate in thoughts to others and what’s the No. 1 reason for quitting smoking, ’cause I want to apply them in my own life.

Many men expressed pride and appreciation when their scores validated and/or directly complimented their parenting skills. A 41-year-old father of 2 showed delight in the “dad score” he received, exclaiming, “I must be a good dad. I got a 5! Woo-hoo! Best thing I’ve heard all night.” This same father explained that the interactive polls and quizzes on the website were “reassuring that, hey, I’m a pretty normal guy. . . . I think some guys like to do the quizzes and sort of see how they match up or measure up, or to see how they’re doing.” Validation that they were capable fathers was viewed as important, as a 25-year-old father of an infant explained:

That was a good reinforcement to let you know that you’re probably doing better than you thought you were. . . . Like, for me, I wanna be a good dad. But you don’t really know [how you are doing], because there’s no real, like, grade, or no real landmarks, or no real milestones that say you are a good dad or a bad dad.

Another man, a lone parent more confident in his fathering skills than other participants, also stated that the quiz scores provided important affirmation, adding, “I’m 95% sure I’m doing the right thing, but there’s still 5% of doubt.”

### Money Matters: Triggering Masculine Virtues Related to Family Finances

The DIG website component with the most impact was the interactive Smoking Calculator, an SC resource. The calculator prompted men to enter the number of daily cigarettes they smoked and the cost per carton. It then generated the monthly and annual dollar amount they spent on cigarettes. This number never failed to elicit shock at the amount of money they were spending on cigarettes. High expenditures on tobacco were particularly meaningful to men for whom financial success and related achievements (eg, providing for a family, buying a car) were important to fulfilling the breadwinner role.

One participant who smoked 15 cigarettes per day, a 23-year-old father of 2 young children, reacted strongly to estimates provided by the calculator by exclaiming loudly, “That is disgusting! Almost $6,000 [CAD] a year! On cigarettes! Holy crap! That makes me sick to my stomach! That’s like double what I thought I spent!” Similarly, a 28-year-old father of 1 child expressed dismay, albeit in a more restrained way, saying, “[I spend] $3,100 [CAD] per year. Staggering. . . . Like, that’s your money. That’s a lot of money.” This new information prompted many to reflect on the benefits of quitting smoking. A few participants found the calculator so persuasive they suggested that it should have a more prominent placement on the DIG website.

### Masculine Ideals: Father Role Models As Cessation Aids

The Smoke Free Dads section of the website offered video testimonials from real-life fathers who had quit smoking. The most popular testimonial, David’s Story, featured a contemporary, fit-looking father talking about his successful quit and how thinking about his family helped him deal with cravings. The video purposely positioned a focus on fathering and being a father as a successful quit strategy. Most participants watched the video with interest and remarked how David inspired them to think about their own quitting. For example, a 22-year-old father who had smoked for 10 years described how he connected to David as an authentic role model:

I just watched a video of Dave talking about quitting smoking . . . it’s pretty heart-warming. And I think that’s one of the videos that will help me, encourage me to quit smoking. Because I was raised by family that smoked while I was younger, too, and that might be 1 of the reasons why I smoke. So, I don’t want my daughter to start smoking because she sees me smoking.

A 33-year-old father who had smoked for 20 years confirmed that he found the notion of using fatherhood as a cessation aid a novel approach:

. . . [B]ecause the less time I spend smoking, the more time I’m gonna be spending with my kid, right? I think it’s good, I’ve never seen it before, so I’m gonna try it . . . just supporting the idea of getting out and doing things with your children instead of smoking’s pretty big.

The few participants who dismissed or rejected David as credible espoused more traditional masculine ideals and presented themselves as committed smokers. These men made it clear that they could not identify with David or contemporary discourses of involved fathering. For example, the 55-year-old father of 5 children stated:

I think there’s a big misconception about dads. And this stuff with them lying in the park, playing with the kids all the time. Going back a million years, dads go out, make the money, bring it home . . . the mammoth, or whatever they’re cooking that night. Right. And the mums do all this [child care] stuff.

A 37-year-old father of 2 who had smoked for 20 years dismissed the Tips on Fathering video by stating that he couldn’t trust a man wearing an earring and “sounding like a hippie,” thereby distancing himself from such masculine tropes. Other men who rejected David’s Story or the underlying relational approach reflected in the website used the argument that the videos or website lacked “hard facts” or new, helpful strategies for quitting. Curiously, the medical facts about smoking and cancer did not appear to threaten their current smoking practices.

### Manly Moves: Physical Activity for the Male Body

The exercise videos and fitness poll components evoked the widest range of responses from the men. These website components focused on the importance of regular physical activity to men’s health and as an aid to cessation, and the components were intended to prompt men to consider how they could integrate physical activity with their responsibilities as fathers. For example, in one video a father pushes a stroller with his infant through the park and does step-up exercises on bleachers while his baby sleeps. Although most of the men presented themselves as sensitive and sensible fathers, when it came to exercise, stereotypical masculine gender norms that frame men as strong and tough trumped their responses. Several men mocked the video, which demonstrated how fathers could exercise with their baby in tow, and criticized the video for portraying exercise that was not vigorous enough. A 43-year-old father of 3 who had quit smoking scorned the video as “too easy” saying, “I dunno if I’d consider it a workout, ’cause this is just everyday exercise that you do with your children.” The baby stroller in the video may have tested the degree to which men could relate to the content, suggesting that strollers and workouts in the same frame were not compatible with the types of physical exertion that provide opportunities for men to challenge themselves.

The suggestions men gave for enhancing the physical activity components of the website highlighted the desire for toughness, competition, and physical performance—all of which align to masculine ideals about what constitutes exercise. A 25-year-old father, while applauding the wide range of workout ideas on the website, stated that for him, exercise was synonymous with lifting weights or using weight machines. Overall, the men were less interested in aerobic workouts such as running or cycling, and instead focused on building muscular strength. A 34-year-old father said, “Yeah, I don’t know if I’d do this. I’d rather just use weights.”

Several men were uninterested in physical activity of any kind and distanced themselves from prescribed workouts with performance evaluations and outcomes. A 37-year-old father of 2, self-described as “lazy,” said that listening to a fit man tell him how to work out just “pisses me off.” This father refuted the legitimacy or motivating influences of such “coaching” or the need to perform physically to claim prowess. Although physical activity to promote cardiovascular fitness was the hardest sell of all the DIG components, it was relevant to a few smokers who were motivated to quit or had already engaged in quitting. For instance, a 33-year-old father of 3, one of the few men who said he wanted to quit for his health, expressed interest in cardio-based workouts. After taking the fitness poll, this father stated that it motivated him because he liked to know “what the going trend is” and “because cardio is something I would like to do, it would encourage me to do it more.”

## Discussion

### Principal Findings

The potential of the Internet to engage men with their own health has been touted as an important antidote to men’s reticence in taking up professional medical services [21]. Indeed, in the context of smoking, stigma exists, rendering many men more likely to deny or conceal their smoking rather than seek “in-person” help toward SC. In addition to providing anonymity, tone and content are lynchpins to engaging men with online SC programs. The current study findings confirmed 3 features as central to online resources for fathers who smoke: (1) a focus on fathering was an effective draw to an SC website for new fathers, (2) nestling masculine virtues of strength and compassion with fathering were conduits for SC among men invested in protecting and providing for their families, and (3) the Internet provided acceptable and accessible avenues for men to find and critique an array of health-promoting strategies that are tangential to and directly target SC. Each of the aforementioned features should also be understood as provisional; some content was taken up, some was dismissed. Indeed, the influence of content varies depending on: (1) the readiness of fathers to take on SC, (2) their buy-in to contemporary fathering discourse and alignments to manly ideals about physical activity, and (3) the believability of our representations of those ideals. In this study we have offered insights into what, as well as why, some content of the DIG website engaged fathers who smoke, but want to quit.

Beyond pretesting, from which the current study findings are drawn, formal, longitudinal evaluations are vital to adjust content and make empirical claims of effectual men’s SC interventions. While Oliffe et al have suggested that Google and YouTube analytics are useful for monitoring the general traffic to men-centered health websites and specific online content [28], there is a need to provide greater empirical assurances about the tangible benefits derived by end-users. Based on the findings from the current study, we suggest 2 key considerations in designing evaluation strategies for men’s Web-based SC interventions. First, many SC interventions are judged entirely on their ability to deliver successful quits. However, interventions are often focused on pre-contemplative and contemplative stages of change in the hope of driving men’s preparation and actions toward the maintenance of behavior change—or in our specific context, sustained SC [29]. In this regard, the expectations and, therefore, the evaluation criteria should be adjusted to capture the stage of change as a means to more reasonably report the impact of specific content and Web pedagogies. Second, it is important to recognize the great diversity that exists within the category of fathers and how this influences the uptake of online SC resources. Expanding the resources to allow users to meet their specific needs/preferences and address a range of masculinities is therefore a key element. Rather than espousing a one-size-fits-all SC intervention, the current study findings demonstrate the usefulness of a multipronged approach to resonate with the diverse masculine ideals embodied by fathers.

### Conclusions

The current study findings add to the nascent body of knowledge about how becoming and being a father represents an opportunity to engage men in SC. Moreover, offered here are some insights for how that might be achieved online. In accord with previous research distilling men’s health promotion program principles [4], Web platforms can provide mechanisms for engaging fathers in SC. The challenge remains to better understand and account for end-user outcomes and thoughtfully consider how best to do virtual SC programs as well as other types of cancer prevention programs for men.

## Abbreviations

DIG: Dads in Gear
SC: Smoking cessation
